# Hantavirus Disease Cluster Caused by Seoul Virus, Germany

**DOI:** 10.3201/eid3001.230855

**Published:** 2024-01

**Authors:** Jörg Hofmann, Rainer G. Ulrich, Calvin Mehl, Stephan Drewes, Jutta Esser, Martin Loyen, Heinz Zeichhardt, Konrad Schoppmeyer, Lioba Essen, Wolfgang Güthoff, Detlev H. Krüger

**Affiliations:** Charité–Universitätsmedizin Berlin, Berlin, Germany (J. Hofmann, D.H. Kruger);; German Centre for Infection Research (DZIF), Partner Site Hamburg-Lübeck-Borstel-Riems, Greifswald-Insel Riems, Germany (R.G. Ulrich, C. Mehl);; Friedrich-Loeffler-Institut, Greifswald-Insel Riems (R.G. Ulrich, C. Mehl, S. Drewes);; Laborarztpraxis Osnabrück, Georgsmarienhütte, Germany (J. Esser);; Herz-Jesu Krankenhaus, Münster-Hiltrup, Germany (M. Loyen);; INSTAND e.V., Society for Promoting Quality Assurance in Medical Laboratories, Düsseldorf, Germany (H. Zeichhardt);; Institut für Qualitätssicherung in der Virusdiagnostik, Berlin (H. Zeichhardt);; Euregio-Klinik, Nordhorn, Germany (K. Schoppmeyer);; Maria-Josef-Hospital Greven, Greven, Germany (L. Essen);; Gesellschaft für Biotechnologische Diagnostik mbH, Berlin (W. Güthoff)

**Keywords:** hantavirus disease, viruses, rat, Seoul virus, Seoul virus diagnostic, zoonoses, Germany

## Abstract

A cluster of 3 persons in Germany experienced hantavirus disease with renal insufficiency. Reverse transcription PCR–based genotyping revealed infection by Seoul hantavirus transmitted from pet rats. Seoul virus could be responsible for disease clusters in Europe, and infected pet rats should be considered a health threat.

Hantavirus disease occurs worldwide. Estimated number of cases is ≈100,000 annually; most cases are in Asia and Europe. Pathogenic hantaviruses are transmitted to humans through excreta of infected rodents. The infection can lead to renal or cardiopulmonary failure; case-fatality rates are up to 50% (*1*). Seoul virus (SEOV) is a hantavirus species for which rats (*Rattus* spp.) are natural hosts. In humans, SEOV infection causes mild to moderate disease with fever, acute kidney injury, hepatitis, and gastroenteritis; it is associated with transient thrombocytopenia and proteinuria (*2*–*4*).

Despite numerous clinical cases and local outbreaks of SEOV infection in Asia, few human cases are known in Europe (*4*–*6*). However, SEOV infections are difficult to identify by routine serodiagnosis because of high cross-reactivity with antigens of related hantaviruses, such as Hantaan virus and Dobrava-Belgrade virus. Whereas Hantaan virus is endemic in Asia, Dobrava-Belgrade virus is found in many parts of Europe (*1*).

Autochthonous human SEOV infection in Germany was described in 2020; the hantavirus type was determined by molecular analysis, and infection source was identified as a pet rat (*4*). Deeper molecular analysis found SEOV strains in several pet rats, including the animals owned by that patient (*7*). However, no clusters of human SEOV infections were reported previously from Germany or any other part of Europe.

## The Study

During November–December 2021, three persons in Germany experienced typical initial signs of hantavirus disease (fever, malaise, myalgia, chills, low-back pain, nausea) (Table 1). The patients lived ≈25 km apart. All 3 patients had acute kidney injury with reduction of glomerular filtration rate, proteinuria, and microhematuria. Moreover, biochemical analysis revealed typical thrombocytopenia and signs of inflammation, including elevation of C-reactive protein and transaminases. Serum creatinine levels remained within reference ranges or were only transiently higher. The patients recovered and were discharged after 3–6 days (Table 1).

**Table 1:** Clinical characteristics of patients infected with Seoul virus, Germany*

Criteria	Patient 1	Patient 2	Patient 3
Domicile	North Rhine-Westphalia	North Rhine-Westphalia	Lower Saxony
Sex	M	F	M
Age, y	31	31	20
Duration of hospitalization, d	3	4	6
Initial fever, °C	40	39	40
Malaise, myalgia, chills, low-back pain, nausea	Y	Y	Y
Serum creatinine elevation	Y†	N	N
Thrombocytopenia	Y	Y†	Y
CRP elevation	Y	Y	Y
Liver enzyme elevation	Y	Y	Y
GFR reduction	Y	Y	N
Proteinuria	Y	Y	Y
Microhematuria	Y	Y	Y
Additional clinical findings	Splenomegaly	Splenomegaly	Lung opacity

*CRP, C-reactive protein; GFR, glomerular filtration rate; LS, Lower Saxony; NRW, North Rhine-Westphalia. †Test result was at the cutoff between positive and negative.

All 3 patients developed IgM and IgG against diagnostic antigens from the group of murine-associated hantaviruses, including Hantaan virus, Dobrava-Belgrade virus, and SEOV. After onset of disease, we performed panhantavirus PCR, reverse transcription PCR based on amplification of a 412-nt region of the genomic large segment (*8*), to detect hantavirus genetic material from serum samples of patients 1 and 3 (Table 2).

**Table 2:** Virus diagnostics results of hantavirus patients and close contacts, Germany*

Person no.	Illness	Pan-hantavirus PCR	Hantavirus IgM†	Hantavirus IgG†
1	Y	+	+	+
2‡	Y	−	+	+
12a§	N	NA	−	−
12b§	N	NA	−	−
3	Y	+	+	+
4¶	N	−	−	+

*NA, not applicable; +, positive; −, negative. †Tested with recomLine HantaPlus IgG and IgM (https://www.mikrogen.de). ‡Wife of patient 1. §Children of patients 1 and 2. ¶Girlfriend of patient 3.

Patients 1 and 2 were married to each other and lived in the federal state of North Rhine-Westphalia. Their children (persons 12a and 12b) (Table 2) remained healthy and did not seroconvert. The family was known to keep pet rats at home. Patient 3, who lived in Lower Saxony, reported that his girlfriend also kept pet rats. Investigation of his girlfriend (person 4) showed that she remained healthy; however, her hantavirus IgM negative/IgG positive serostatus suggested a previous subclinical infection (Table 2).

The family of patients 1 and 2 permitted us to test their pet rats. We amplified the corresponding region from the genomic large segment. Phylogenetic analysis of the amplified virus sequences from patients 1 and 3 and the pet rats revealed almost identical sequences (Figure). We compared them to the amplified virus sequences from a previous unrelated patient and her pet rat from Lower Saxony (*4*). 

**Figure:**
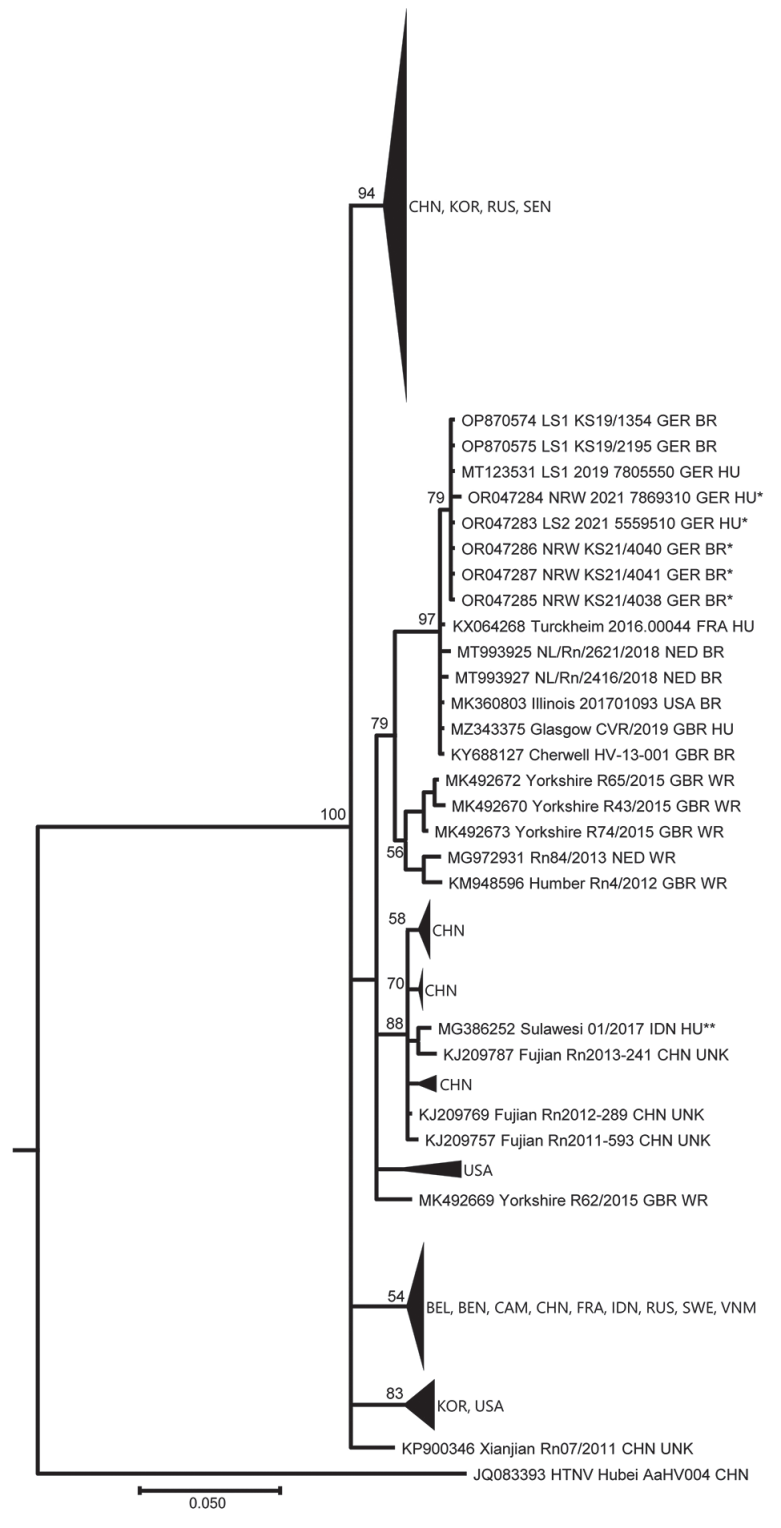
Phylogenetic tree of partial large segment Seoul virus sequences from humans and rats, Germany. Segments were 412-nt long, positions nt 2919–3330 based on reference sequence (KM948594_Cherwell_GBR_BR). The partial large segment Bayesian tree was reconstructed using 20 million generations and the Hasegawa-Kishino-Yano substitution model with gamma distribution and invariant sites. Single asterisks indicate sequences from this study, denoted by their GenBank accession numbers. Double asterisks indicate sequence from the imported Seoul virus case from Indonesia (*9*). Aa, *Apodemus agrarius*; BEL, Belgium; BEN, Benin; BR, breeder rat (includes feeder, lab, and pet rats); CAM, Cambodia; CHN, China; FRA, France; GBR, Great Britain; GER, Germany; HTNV, Hantaan virus; HU, human; IDN, Indonesia; KOR, Korea; L, large segment; NED, the Netherlands; Rn, *Rattus norvegicus*; RUS, Russia; SEN, Senegal; SEOV, Seoul virus; SWE, Sweden; UNK, unknown wild or breeder rat; USA, United States of America; VNM, Vietnam; WR, wild rat.

Phylogenetic analysis disclosed the detected virus sequences as SEOV and, moreover, demonstrated the high relatedness of virus sequences from patient and pet rats from North-West Germany. The high similarity of SEOV sequences from Germany to sequences derived from breeder rats in the Netherlands, France, and the United States, but not to sequences from wild rats of those countries, suggests an intensive exchange of pet rats between neighboring countries in Europe and between Europe and the United States (*7*).

Human SEOV infections might be underestimated because the nucleocapsid proteins used in serologic assays share high amino acid sequence similarity and cross-reactivity with different hantavirus species. To explore this hypothesis in an external quality assessment, we sent a serum sample from patient 3, obtained 2 months after complete recovery, to 8 expert laboratories in Europe. All laboratories analyzed the sample in their routine hantavirus diagnostics, including commercial and lab-derived enzyme immunoassays, immunofluorescence assays, rapid assays, and immunoblots for confirmation (Appendix Tables 1, 2). Results revealed strong cross-reactivities to related hantavirus nucleocapsid proteins in IgG and IgM screening and in confirmation assays, making correct serotyping impossible. Thus, in cases of unexpected pattern in serodiagnostics or cases of known contact to rats, we strongly recommend verifying positive results with molecular methods or by typing of neutralizing antibodies. Only the laboratory using a focus reduction neutralization assay identified the SEOV infection (Appendix Table 1).

## Conclusions

A total of 6 molecularly proven SEOV-infected patients in Germany have been described: 1 imported case (*9*), 1 autochthonous case (*4*), and the 3 symptomatic case-patients and 1 asymptomatic case-patient we described in this report. Five of these persons had been admitted to hospital for several days. In all autochthonous cases, pet rats were confirmed as source of infection, strongly suggesting a need for close cooperation between public health and animal health institutions in the One Health frame. Pet rats, in addition to wild and breeder or feeder rats, should be considered threats for SEOV infection in humans.

Appendix Additional information about a cluster of hantavirus disease cases caused by Seoul virus, Germany.
