# Supplementary material for: Hantavirus Disease Cluster Caused by Seoul Virus, Germany
Source: Emerg Infect Dis. 2024 Jan;30(1):133–5. doi: 10.3201/eid3001.230855 (PMC10756357; doi:10.3201/eid3001.230855)
Supplement: Appendix — Additional information about a cluster of hantavirus disease cases caused by Seoul virus, Germany. [file 23-0855-Techapp-s1.pdf]

Article DOI: http://doi.org/10.3201/eid3001.230855

*EID cannot ensure accessibility for supplementary materials supplied by authors. Readers who have difficulty accessing supplementary content should contact the authors for assistance.*

# Hantavirus Disease Cluster Caused by Seoul Virus, Germany

## Appendix

**Appendix Table 1.** Results of the hantavirus routine diagnostics performed in 8 expert laboratories in Europe*

| Laboratory | Routine assay used | Result IgG | Result IgM | Interpretation made by the laboratory |
|---|---|---|---|---|
| A | Hantavirus Hantaan virus IgG/IgM EIA | pos | pos | acute hantavirus infection without typing |
| | Hantavirus Puumala virus IgG/IgM EIA | pos | pos | |
| B | Hantavirus-Mosaic 2 IgG and IgM IFA | dilution 1:10,000 HTNV | dilution 1:100 SEOV | no typing possible |
| C | cFRNT in house (IgG only) | PUUV (1:173), DOBV (1:147), HTNV (1:890), SEOV (1:1648) | | evidence for SEOV infection |
| D | Reagena Puumala virus IgG/IgM | neg | neg | no hantavirus infection |
| E | In-house IFA Dobrava-Belgrade virus IgG/IgM | 1:5,120 | 1:320 | no typing possible |
| | In-house IFA Puumala virus IgG/IgM | 1:320 | < 1:20 | |
| | In-house IFA Seoul virus IgG/IgM | 1:5,120 | 1:80 | |
| | In-house IFA Hantaan virus IgG/IgM | 1:5,120 | 1:320 | |
| F | Hantavirus DxSelect IgG/IgM EIA | pos (s/c ratio: 7.54) | pos (s/c ratio: 7.41) | acute hantavirus infection without typing |
| G | Eurasia Hantavirus pool 1 EIA | pos | pos | acute hantavirus infection without typing |
| H | Hantavirus profile 1 EIA | pos | pos | past DOBV infection |

The serum sample was obtained from patient #3 approx. two months after complete recovery from his Seoul virus (SEOV) infection. cFRNT, chemiluminscence focus reduction neutralization assay; IFA, immunofluorescence assay; EIA, enzyme immunoassay; pos, positive; HTNV, Hantaan virus; PUUV, Puumala virus; DOBV, Dobrava-Belgrade virus; SEOV, Seoul virus
Note: Although different screening assays were used, no single assay (except cFRNT) allowed a definitive typing. SEOV infection may be overseen if hantavirus screening is done only with a PUUV rapid assay (see laboratory D).
Results were generated in the laboratories headed by Dres Baillot, Bohnert, Eis-Hübinger, Emmerich, Enders, Ganzenmüller, Maes, Schüttler and our own (alphabetical order, not corresponding with designation laboratory A-H)

**Appendix Table 2.** Results of hantavirus confirmation assays performed on a serum sample from patient 3 in the expert laboratories*

| | | *recom*Line HantaPlus IgG and IgM (Mikrogen) | | | | |
|---|---|---|---|---|---|---|
| Laboratory | Test | PUUV | SNV | HTNV | DOBV | SEOV |
| A | IgG | +/- | - | +++ | +++ | + |
| | IgM | +++ | ++ | +++ | +++ | ++ |
| B | IgG | +/- | - | ++ | ++ | + |
| | IgM | ++ | ++ | ++ | ++ | ++ |
| C | IgG | - | - | + | + | + |
| | IgM | + | + | + | + | + |
| D | IgG | +/- | - | ++ | ++ | + |
| | IgM | + | + | + | + | +/- |
| E | IgG | +/- | - | ++ | ++ | ++ |
| | IgM | ++ | ++ | ++ | ++ | ++ |
| F | IgG | + | - | +++ | +++ | ++ |
| | IgM | ++ | ++ | ++ | ++ | ++ |
| I | IgG | +/- | - | +++ | +++ | + |

| Laboratory | Test | *recom*Line HantaPlus IgG and IgM (Mikrogen) PUUV | SNV | HTNV | DOBV | SEOV |
|---|---|---|---|---|---|---|
| | IgM | +++ | ++ | +++ | +++ | + |
| | | Hantavirus Profil 1 IgG and IgM (Euroimmun) | | | | |
| | | PUUV | SNV | HTNV | DOBV | SEOV |
| G | IgG | ++ | - | +++ | +++ | n.d. |
| | IgM | +/- | - | +/- | +/- | n.d. |

*PUUV, Puumala virus; SNV, Sin Nombre virus; HTNV, Hantaan virus; DOBV, Dobrava-Belgrade virus; SEOV, Seoul virus

Note: All laboratories observed cross-reactivity even on PUUV and SNV nucleocapsid proteins in the IgM assay and less pronounced in the IgG-assay. The data clearly demonstrate, that SEOV cannot be identified as the cause of the SEOV infection.

Results were generated in the laboratories headed by Dres Baillot, Bohnert, Eis-Hübinger, Emmerich, Enders, Ganzenmüller, Maes, Schüttler and our own (alphabetical order, not corresponding with designation laboratory A-H).
